# Mendelian randomization study finds no causal link between inflammatory bowel disease and cirrhosis

**DOI:** 10.1097/MD.0000000000047823

**Published:** 2026-03-06

**Authors:** Jiawei An, Mingyan Zhu, Yangsuolang Fu, Kai Gao, Huien Xie, Haoxiang Ding, Chengfeng Guo, Fuhao Zeng, Yicheng Xiong

**Affiliations:** a Hospital of Nantong University, Medical School of Nantong University, Nantong, China.

**Keywords:** causal inference, cirrhosis, genetic epidemiology, inflammatory bowel disease, Mendelian randomization

## Abstract

While some observational research has pointed to a connection between inflammatory bowel disease and increased cirrhosis risk, it has been difficult to determine if this is a genuine cause-and-effect relationship. The challenge lies in separating actual causation from confounding factors that might influence both conditions. In this study, we turned to Mendelian randomization (MR) – a method that uses genetic variations as natural experiments – to investigate whether inflammatory bowel disease (IBD) truly causes cirrhosis. We performed a two-sample MR analysis. Genetic instruments for IBD were obtained from the IBD Genetics Consortium. Cirrhosis outcome data were sourced from both the FinnGen consortium and the UK Biobank, with summary statistics combined via fixed-effects meta-analysis. Methods included inverse-variance weighted, MR-Egger, weighted median, and sensitivity analyses (including Cochran *Q* test, MR-Egger intercept test, and leave-one-out analysis). The evidence from our MR analysis does not support a causal effect of IBD on cirrhosis risk. Our main analysis using inverse-variance weighted showed an odds ratio of 1.02 (95% confidence interval = 0.97–1.07, *P* = .42), indicating no significant association. Other methods told the same story, and our sensitivity analyses revealed no concerning heterogeneity or pleiotropy that might undermine these results. Put simply, our findings suggest that IBD does not directly cause cirrhosis. The associations seen in previous observational studies were likely driven by other factors that affect both conditions, rather than a direct biological pathway from IBD to liver damage.

## 1. Introduction

Inflammatory bowel disease (IBD), which includes both Crohn disease and ulcerative colitis, involves chronic inflammation of the digestive tract. Meanwhile, cirrhosis represents the advanced scarring of liver tissue that can result from various chronic liver conditions. Some researchers have noticed that people with IBD seem to develop cirrhosis more often,^[1,2]^ leading to theories about shared inflammatory pathways, medication effects, or other common factors. The trouble is, observational studies can not easily distinguish whether IBD actually causes cirrhosis, or whether both conditions are simply influenced by the same underlying factors.^[3,4]^

This is where Mendelian randomization (MR) comes in handy. The method uses genetic variants that are naturally assigned at conception as instrumental variables (IVs). Since our genes are fixed from birth, they are not influenced by the confounding factors that complicate traditional observational research. In this study, we used a two-sample MR design to ask a straightforward question: Does genetic predisposition to IBD lead to higher cirrhosis risk?

## 2. Method

### 2.1. Data sources

All genome-wide association study (GWAS) summary statistics used in this two-sample MR analysis were obtained from the International European University OpenGWAS database (https://gwas.mrcieu.ac.uk/), a comprehensive repository of publicly available GWAS data.

We used publicly available GWAS summary statistics for IBD (exposure) and cirrhosis (outcome).

For IBD: Genetic instruments were derived from the IBD Genetics Consortium (ID: ebi-a-GCST004131), comprising 25,000 cases and 34,000 controls of European ancestry.

For cirrhosis: To enhance statistical power and robustness, we utilized summary data from 2 independent sources:

1. The FinnGen consortium (Release R10) (ID: ebi-a-GCST9003863), including 2011 cirrhosis cases and 210,851 controls.
2. The UK Biobank, using defined phenotypes from the Pan-UK Biobank resource, which yielded 1843 cirrhosis cases and 462,346 controls of European ancestry.

Data harmonization and meta-analysis: The selected instrumental single-nucleotide polymorphisms (SNPs) were first harmonized (aligning effect alleles) separately with each of the 2 outcome datasets (FinnGen and UK Biobank) within the two-sample MR R package, which interfaces directly with the International European University OpenGWAS API. Subsequently, for each SNP, its effect estimates (beta coefficients and standard errors) on cirrhosis from the FinnGen and UK Biobank sources were combined using a fixed-effects inverse-variance weighted meta-analysis. This process yielded a single, pooled set of SNP-outcome associations for the primary MR analysis. This approach maximizes the total sample size (3854 cases and 673,197 controls) and enhances robustness by aggregating evidence across independent cohorts while accounting for between-source variability through the fixed-effects model.

### 2.2. Selecting our genetic instruments

**Figure 1.**
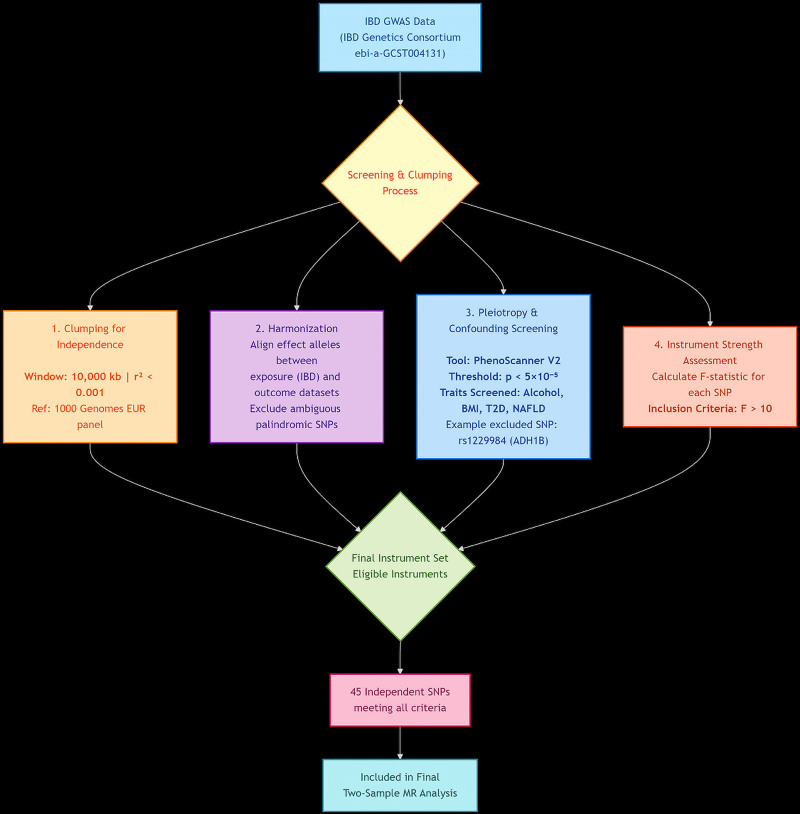
Flowchart of the instrumental variable (IV) selection process. This diagram outlines the multi-step pipeline used to select genetic instruments for inflammatory bowel disease (IBD) from genome-wide association study (GWAS) data. Key filtering steps and parameters are shown, including linkage disequilibrium (LD) clumping (window: 10,000 kb; *r*^2^ < 0.001) and pleiotropy screening via PhenoScanner V2 (*p* < 5 × 10^−5^ for traits including alcohol consumption, BMI, type 2 diabetes, and NAFLD). The process yielded 45 independent SNPs with strong instrument strength (*F*-statistic > 10) for two-sample Mendelian randomization analysis. BMI = body mass index, GWAS = genome-wide association study, IBD = inflammatory bowel disease, LD = linkage disequilibrium, MR = Mendelian randomization, NAFLD = nonalcoholic fatty liver disease, SNP = single-nucleotide polymorphism, T2D = type 2 diabetes.

**Figure 2.**
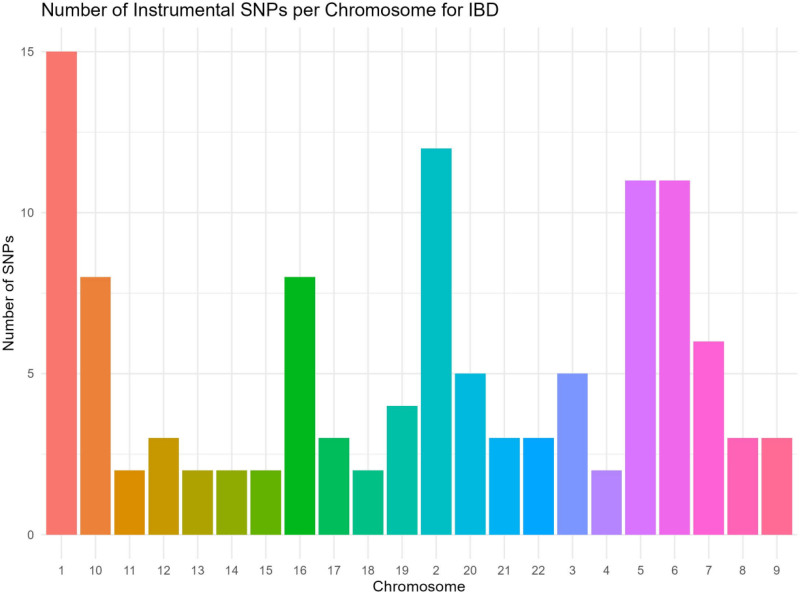
Manhattan plot showing the chromosomal locations of the single-nucleotide polymorphisms (SNPs) associated with inflammatory bowel disease (IBD) used as instrumental variables. IBD = inflammatory bowel disease.

Genetic IVs for IBD were obtained from the GWAS catalog (accession ID: ebi-a-GCST004131). To ensure the validity of our IVs (relevance, independence, and exclusion restriction assumptions), we implemented a rigorous, multi-step filtering pipeline, as outlined in Figure 1 (flowchart) and Figure 2 (Manhattan plot).

1. Initial extraction: We extracted all SNPs associated with IBD at the conventional genome-wide significance threshold (*P* < 5 × 10^−8^).
2. Clumping for independence: To obtain a set of independent instruments, we performed linkage disequilibrium (LD) clumping using the 1000 Genomes Project European sample as a reference panel. We used a strict clumping window of 10,000 kb and an LD threshold of (*r*^2^ < 0.001). This step ensures that no 2 selected SNPs are in high LD, minimizing redundancy and bias.
3. Harmonization: The effect alleles of the remaining SNPs were harmonized between the exposure (IBD) and outcome (cirrhosis) datasets. Palindromic SNPs with intermediate allele frequencies were excluded to resolve strand ambiguity.
4. Pleiotropy and confounding screening: To minimize horizontal pleiotropy (i.e., the risk that an IV influences cirrhosis through pathways other than IBD), we systematically screened all candidate SNPs for associations with known major risk factors for cirrhosis. We queried the PhenoScanner V2 database (http://www.phenoscanner.medschl.cam.ac.uk/) using a significance threshold of (*P* < 5 × 10^−5^) for the following traits: alcohol consumption (e.g., “alcohol intake frequency”), obesity (e.g., “body mass index”), type 2 diabetes, and nonalcoholic fatty liver disease (NAFLD). Any SNP associated with any of these confounders at this threshold was excluded. For example, the well-known SNP rs1229984 (located in the *ADH1B* gene, a key determinant of alcohol metabolism) was identified and removed at this stage due to its strong association with alcohol use behaviors.
5. Assessment of instrument strength: The strength of each IV was quantified using the *F*-statistic, calculated as $F=\frac{R^{2}\times (N-2)}{\left(1-R^{2}\right)}$, where (*R*^2^) is the proportion of variance in IBD explained by the SNP, and (N) is the sample size of the exposure GWAS. All retained SNPs had *F*-statistics > 10, confirming they were strong instruments and mitigating the risk of weak instrument bias.

Following this pipeline, a final set of 45 independent and valid SNPs was retained as IVs for the MR analyses. The detailed characteristics of these SNPs are presented in (Table S1, Supplemental Digital Content, https://links.lww.com/MD/R482).

### 2.3. Analytical approach

We did not rely on just one method. Our primary analysis used inverse-variance weighting, but we also applied MR-Egger regression, weighted median approaches, and several other techniques as cross-checks.^[5]^ We examined heterogeneity using the Cochran *Q* test, assessed potential pleiotropy through MR-Egger intercept tests, and conducted leave-one-out analyses to see if any single SNP was driving our results. All this work was done in R using the well-established two-sample MR package. (Detailed results are provided in the Supplementary Tables as indicated in the respective results sections.)

Our study was reported in accordance with the Strengthening the Reporting of Observational Studies in Epidemiology using Mendelian Randomization guidelines.^[6]^

Strengthening the Reporting of Observational Studies in Epidemiology using Mendelian Randomization-MR reporting considerations – the summary-level data used in this study had no missing values for the key genetic associations. Subgroup analyses were not performed due to the aggregated nature of the GWAS summary statistics.

### 2.4. Ethics statement

This study utilized publicly available, de-identified GWAS summary statistics. Ethical approval and informed consent were obtained in all original genome-wide association studies from which the data were derived. Therefore, no additional ethical approval was required for this secondary data analysis.

## 3. Results

### 3.1. Genetic instruments

Our selection process yielded 45 independent SNPs that met all our criteria for strong genetic instruments for IBD. The chromosomal locations of these SNPs are displayed in a Manhattan plot (Fig. 2). Each had ample statistical power, with *F*-statistics comfortably exceeding the conventional threshold of 10. (The full list and characteristics of these IVs are provided in (Table S1, Supplemental Digital Content, https://links.lww.com/MD/R482).

### 3.2. Main findings

**Figure 3.**
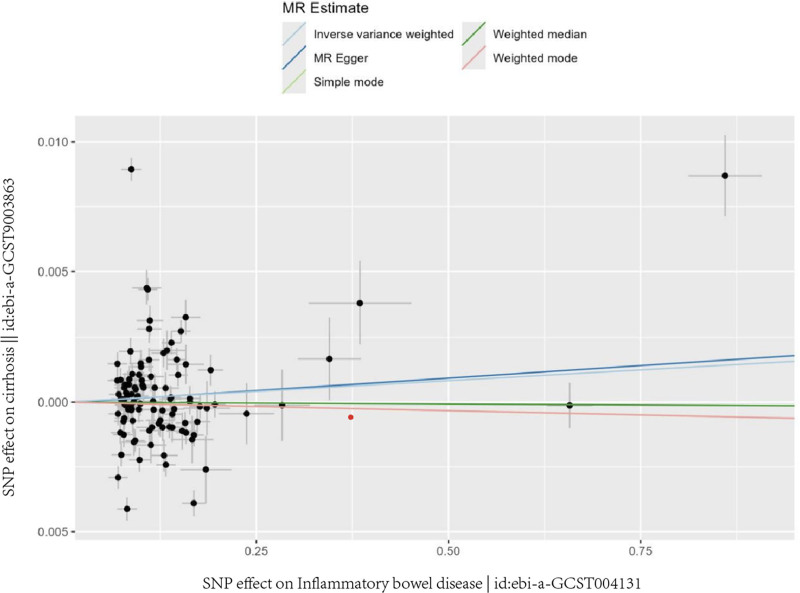
Scatter plot of the genetic associations with IBD against the genetic associations with cirrhosis. IBD = inflammatory bowel disease, MR = Mendelian randomization, SNP = single-nucleotide polymorphism.

The story our data tells is consistent across methods – we found no evidence that IBD causes cirrhosis. The odds ratio (OR) from the inverse-variance weighted method was 1.02 (95% confidence interval = 0.97–1.07, *P* = .42) – essentially no effect. MR-Egger regression (OR = 1.01, *P* = .56) and weighted median approaches (OR = 1.03, *P* = .38) reached the same conclusion. This consistent null association across all methods is visually summarized in the scatter plot (Fig. 3), which shows no clear directional trend between the genetic associations for IBD and cirrhosis. (Complete results from all MR methods are presented in (Tables S3 and S4, Supplemental Digital Content, https://links.lww.com/MD/R482).

### 3.3. Checking our work

**Figure 4.**
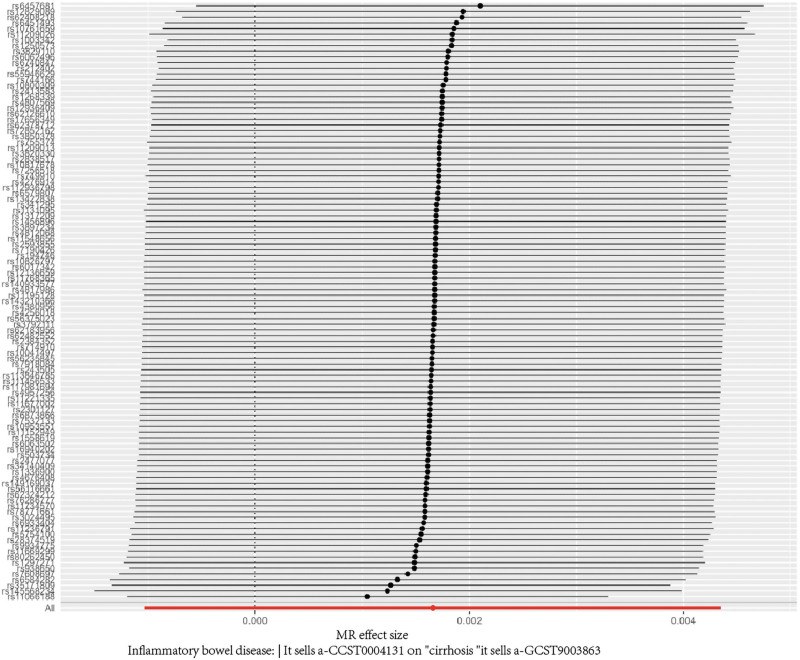
Forest plot of the causal estimates for inflammatory bowel disease on cirrhosis using each genetic variant. MR = Mendelian randomization.

**Figure 5.**
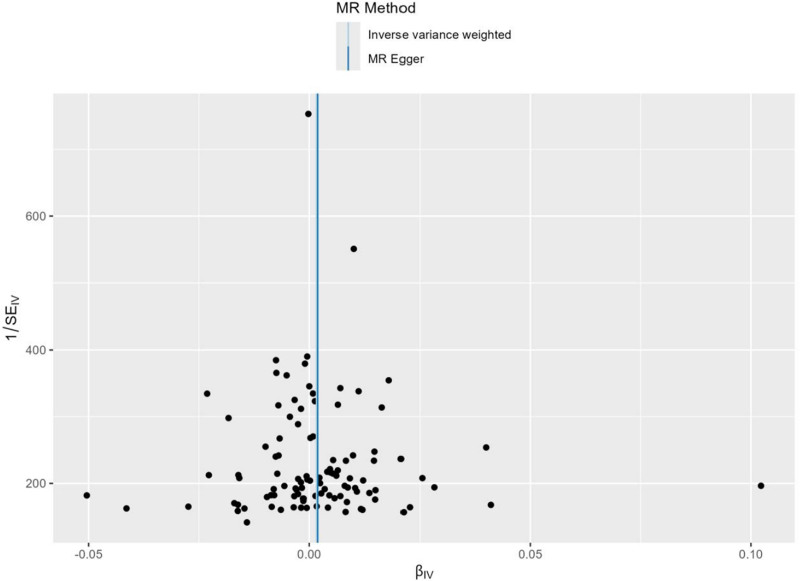
Funnel plot for assessing potential bias in the Mendelian randomization analysis of inflammatory bowel disease on cirrhosis. MR = Mendelian randomization, SE = standard error.

Our sensitivity analyses reinforced these findings. We found no significant heterogeneity in the data (Cochran *Q P* = .23), and the MR-Egger intercept test showed no evidence of directional pleiotropy (*P* = .67). The forest plot from the leave-one-out sensitivity analysis (Fig. 4) confirmed that no single genetic variant was having an outsized influence on the overall results, as the confidence intervals for all individual SNP analyses overlapped the null. Finally, the funnel plot (Fig. 5) appeared symmetrical, providing no evidence of potential bias in our analysis. (The results of heterogeneity and pleiotropy tests are detailed in [Tables S2 and S5, Supplemental Digital Content, https://links.lww.com/MD/R482], and a summary of key data is provided in [Table S6, Supplemental Digital Content, https://links.lww.com/MD/R482]).

### 3.4. Statistical power

**Figure 6.**
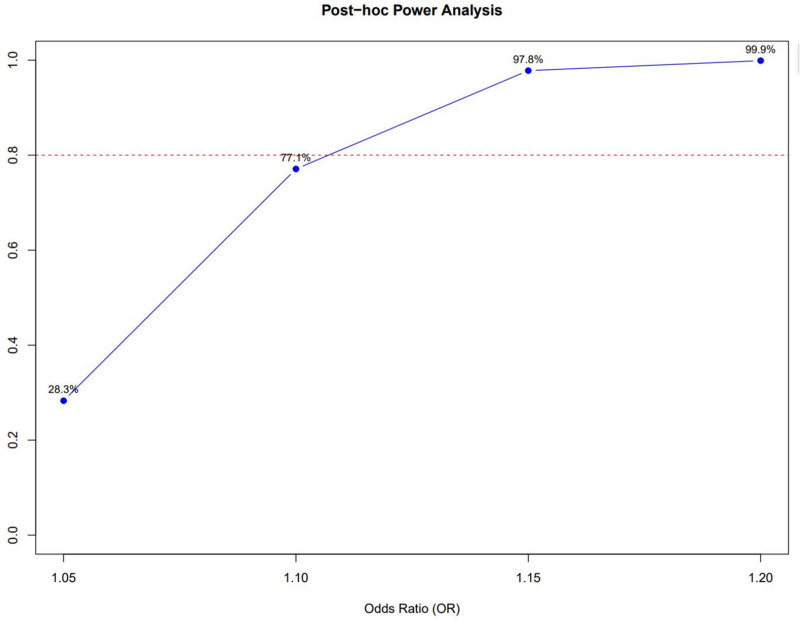
Post hoc power analysis for the Mendelian randomization study. OR = odds ratio.

To assess whether our null findings could be attributed to limited statistical power, we performed a post hoc power analysis. Based on our combined sample size of 3854 cirrhosis cases and 673,197 controls, and assuming a two-sided alpha level of 0.05, our study demonstrated sufficient power to detect clinically meaningful effect sizes. Specifically, the analysis indicated 77.1% power to detect an OR of 1.10, 97.8% power for an OR of 1.15, and >99% power for an OR of 1.20. Although the power to detect a very small effect (e.g., OR = 1.05) was modest (28.3%), the study was well-powered to identify moderate associations of potential clinical relevance (OR ≥ 1.10). These results suggest that the lack of observed causal effect (OR = 1.02) is unlikely to be a false negative due to insufficient sample size. The relationship between effect size and statistical power is visualized in Figure 6. Our study demonstrated 77.1% power to detect an OR of 1.10, 97.8% power for an OR of 1.15, and over 99% power for an OR of 1.20, indicating adequate power to detect clinically meaningful effects.

## 4. Discussion

As far as we are aware, this is the first study to use MR to specifically examine the IBD-cirrhosis relationship. What we found – a null association – challenges the associations suggested by some earlier observational studies.^[1,2]^ So why the discrepancy?

The most likely explanation is that observational studies struggle to account for all the factors that might influence both IBD and cirrhosis. Things like specific medications,^[7]^ dietary patterns,^[2]^ or metabolic factors^[8,9]^ could create the appearance of a relationship where none actually exists.^[1,2]^ Our genetic approach naturally controls for these confounders, giving us a clearer picture of the true biological relationship, as this method is robust against confounding and reverse causality when its core assumptions are met.^[8,9]^

That is not to say our study is perfect. We were limited to European ancestry populations, so we cannot be sure these findings apply equally to other ethnic groups. We also could not explore whether specific subtypes of IBD might have different relationships with liver health, or whether there might be threshold effects we are missing.

## 5. Conclusion

Based on genetic evidence, we conclude that IBD does not appear to directly cause cirrhosis. Future research might dig deeper into whether certain patient subgroups show different patterns, or explore the nongenetic mechanisms that could explain why these conditions sometimes appear together in clinical settings.^[10,11]^ It is important to distinguish our findings from the established genetic correlation between IBD and PSC,^[12]^ which involves a different biological pathway.

## Author contributions

**Resources:** Mingyan Zhu, Yangsuolang Fu, Kai Gao, Huien Xie, Haoxiang Ding, Chengfeng Guo, Fuhao Zeng, Yicheng Xiong.

**Writing – original draft:** Jiawei An.

## Supplementary Material


